# Psychosocial correlates of using faith healing services in Riyadh, Saudi Arabia: a comparative cross-sectional study

**DOI:** 10.1186/1752-4458-9-8

**Published:** 2015-01-28

**Authors:** Fahad D Alosaimi, Youssef Alshehri, Ibrahim Alfraih, Ayedh Alghamdi, Saleh Aldahash, Haifa Alkhuzayem, Haneen Al-Beeshi

**Affiliations:** Department of Psychiatry, King Saud University, P.O. Box 7805, Riyadh, 11472 Kingdom of Saudi Arabia; Department of Psychiatry, Prince Sultan Medical Military City, Riyadh, Saudi Arabia; Psychiatry Resident, Saudi Commission for Health Specialties, Riyadh, Saudi Arabia

**Keywords:** Faith healing, Psychosocial correlates, Prevalence, Psychiatric disorders, Saudi Arabia

## Abstract

**Background:**

In this study, we compared the prevalence of psychiatric disorders and the characteristics of those who either use or do not use faith healers (FHs) services. We also assessed the independent factors of study subjects associated with using FHs.

**Methods:**

This cross-sectional study compared those who use FHs (n = 383) with a control group of those who do not use them (i.e., shopping mall visitors, n = 424) using a survey of sociodemographic characteristics and a validated Arabic version of the Mini International Neuropsychiatric Interview (MINI 6.0).

**Results:**

Participants who ranked higher among FH users included males, people who were either married, divorced, or widowed, those with less education, and those with lower income. They were more likely to report past medical and psychiatric history. Those with diagnosable psychiatric disorders were more likely to visit FHs, especially if the diagnosis was of psychotic and bipolar disorders. The prevalence of psychiatric disorders was higher among FH users, and depressive and anxiety disorders were the most prevalent.

**Conclusions:**

The study showed that having past psychiatric history and a current psychiatric disorder are risk factors for using FHs. Also, a high percentage of FH users had a diagnosable psychiatric disorder. Further research should assess how to facilitate their access to the mental health system.

## Background

Traditional healers represent an undeniable source of care for people with psychiatric disorders in developing countries [1]. Those who most utilize traditional healers are thought to be uneducated, poor, and lacking access to the health care system [2]. However, there is a large number of overlapping social, economic, and cultural factors that determine patient help-seeking behavior from traditional and faith healers (FHs) [3]. The prevailing belief system plays important roles in shaping the use of faith healing from people with psychiatric disorders [4].

Religion plays an important role in the lives of people in Saudi Arabia where Islam is the state religion. Many patients seek the help of FHs for a wide variety of physical and psychiatric problems before turning to modern medicine [5]. The FHs operate in harmony with a shared world view of the society that includes beliefs in magic, evil eye, and possession [6]. The FHs typically perform religious-based practices, such as reading the Holy Quran, as a source of healing. Also, they claim to follow the prophetic traditions of the prophet Mohammad with regard to the health, sickness, and treatment [7].

Psychiatric patients within the faith healing system have not been widely studied. In this study, we compared the prevalence of psychiatric disorders and characteristics of both those who use and do not use FHs. We also assessed the independent factors of study subjects associated with visiting FHs.

## Methods

### Setting

This study was carried out in a number of faith healing settings and shopping malls in Riyadh, the capital city of Saudi Arabia, which has a population of approximately 6 million people.

### Subjects

The study subjects included visitors seeking faith healing from FHs as well as regular shoppers at shopping malls. Those who were 18 years and older and gave consent were included in the study.

### Study design

This cross-sectional survey of FH users (n = 383) included 424 controls who did not use FHs. Given the lack of the data on the prevalence of psychiatric disorders among the general population of Saudi Arabia, our sample size was estimated based on the sample size of a similar study in Sudan [8]. We hypothesized that those with psychiatric disorders were more likely to visit FHs. We also hypothesized that the prevalence of psychiatric disorders among FH users was higher than among non-users. Ethical approval was obtained from the institutional review board at the Faculty of Medicine at King Saud University, Riyadh, Saudi Arabia.

### Study instruments

A questionnaire designed by the authors was used to obtain sociodemographic characteristics of the study subjects, and included age, sex, marital status, education, and monthly income. It also assessed self-reported past medical or psychiatric disorders and whether there was a history of seeking help from FHs. A multi-disciplinary committee with backgrounds in psychiatry, faith healing, and epidemiology validated the content of the questionnaire. The questionnaire was validated in terms of its temporal stability, by using test-retest method on 20 subjects. The Spearman correlation was in the range of 0.55 to 0.70 across all the items and the internal consistency of the questionnaire, by using Cronbach’s α was 0.76.

A validated Arabic version of the Mini International Neuropsychiatric Interview (MINI 6.0) was also administered as a psychiatric diagnostic instrument. The MINI was designed to meet the need for a short but accurate structured psychiatric interview for multicentered clinical trials and epidemiology studies and to be used as a first step in outcome tracking in nonresearch clinical settings. It takes approximately 15 minutes to administer and covers 17 Axis I disorders (i.e., mood, anxiety, substance use, psychotic, and eating disorders), a suicidality module, and one Axis-II disorder (antisocial personality disorder). It has been validated against the much longer Structured Clinical Interview for DSM diagnoses (SCID-P) in English and French and against the Composite International Diagnostic Interview for ICD-10 (CIDI) in English, French, and Arabic [9, 10].

### Data collection

The study was carried out between September 2012 and July 2013. The authors selected the subjects who were readily available in faith healing settings and shopping malls for convenient sampling. Informed written consent was obtained from all participants after explanation of the study objectives. Only four of six FHs in Riyadh allowed us to conduct our studies at their places of business. One refused because he thought we were trying to recruit his patients to our own psychiatric clinics, and another refused without giving any reason. A total of 321 participants were recruited by this means. The controls were recruited from five major shopping malls in Riyadh. Of them, 62 were currently visiting FHs and were therefore added to the study group so that a total of 383 participants were in this group.

### Data analysis

Statisticalanalysis was carried out using SPSS PC+ version 21.0 statistical software (Chicago, USA). Descriptive statistics (i.e., mean, standard deviation, and percentages) were used to quantify the quantitative and categorical variables. The student *t* test was used to compare the means of quantitative variables of the groups. Pearson’s chi-square test and odds ratios were used to test and measure associations between the categorical study outcome variables. Stepwise forward multivariate binary logistic regression was used to identify the independent factors related to the binary outcome variable (i.e., visiting or not visiting FHs) and to obtain its adjusted odds ratio. Statistical significance was recognized when p < 0.05, and 95% confidence intervals were used to report the precision of the estimates.

## Results

Of the total 807 study subjects, 383 (47.4%) from faith healing settings and shopping malls settings have used FHs services. The remaining 424 (52.6%) were those from the shopping malls who had not used FHs services (the control group). The mean (standard deviation) age of these study subjects is 31.5 (10.7). The mean age is statistically significantly higher in users of FHs (34.2 years) than the control group (29.1 years).

Higher proportions of FHs using were found in males (63.2%) and singles (46.2%). The association between these sociodemographic characteristics of those using the FHs in comparison to the control group shows a high statistically significant association. For example, the odds of male study subjects using FHs for their health problems is 3.9 times more than female using FHs. Also, the odds of married and who were either divorced or widowed using FHs is 2.4 times and 3.7 times more compared to study subjects who were single.

About 48.3% of the participants had intermediate or secondary levels of education, and about 40.1% had university degrees. A small percentage had lower educational levels or was illiterate (6.1%). Those who were illiterateor had only a primary level of education, and those who had intermediate or secondary levels of education were more likely to use FHs for their health problems. Their odds ratios of 18.8 and 3.0, respectively, indicate that there is a statistically significant association between these levels of education and using FHs compared to those who held Master's or Ph.D. degrees.

About 65.5% of the participants reported that their monthly income was less than 10,000 Saudi Riyals (SR) or $2,667 US, which is significantly associated with using FHs (Table 1).

**Table 1:** **Distribution of socio-demographic characteristics of study subjects and their associations with use or non-use of faith healers (FHs)**

Variable	No. (%)	FH users (n = 383)	Control group (n = 424)	***t-***value/χ ^2^-value	p-value	95% CI
*Age (years) Mean ± SD*		34.2(11.0)	29.1(9.8)	6.8	<0.0001	(3.6, 6.5)
						**Odds ratio (95% CI)**
*Sex*						
Male	510(63.2)	303(79.1)	207(48.8)	79.4	<0.0001	3.9(2.9, 5.4)
Female	297(36.8)	80(20.9)	217(51.2)			1.0
*Marital Status (n = 381; 420)*						
Single	371(46.2)	131(34.3)	240(57.1)	42.9	<0.0001	1.0
Married	388(48.3)	222(58.4)	166(39.6)			2.4(1.8, 3.3)
Divorced & Widowed	42(5.5)	28(7.3)	14(3.3)			3.7(1.9, 7.2)
*Educational status (n = 381; 422)*						
Illiterate or primary school	49(6.1)	44(11.5)	5(1.2)	101.06	<0.0001	18.8(6.1, 57.9)
Intermediate or Secondary school	388(48.3)	228(59.9)	160(37.9)			3.0(1.6, 5.9)
University or Diploma	322(40.1)	95(24.9)	227(53.8)			0.9(0.4, 1.8)
Master or PhD	44(5.5)	14(3.7)	30(7.1)			1.0
*Monthly Income (n = 275; 257)*						
<10000 SR	337(65.5)	194(70.5)	143(55.6)	12.7	<0.00001	1.9(1.3, 2.7)
> = 10000 SR	195(34.5)	81(29.5)	114(44.4)			1.0

The odds of using FHs by those who reported a history of past medical or psychiatric illness were 2.6 and 5.8 times higher than those who did not report such histories. The prevalence of past medical or psychiatric illness in the study group was 40.5% and 23.2%, respectively, and was significantly higher when compared tothe control group (20.8% and 5%). The prevalence of psychotic, bipolar, depressive, anxiety and other disorders (e.g., alcohol- and substance- related disorders or eating disorders) in the study group were significantly higher than in the control group. The odds ratios of these disorders indicate a significant association between the presence of these disorders and use of FHs. The odds ratios were 27.0 for psychotic disorder, 7.7 for bipolar disorder, 2.9 for depressive disorder, 2.2 for anxiety disorder, and 3.3 for other disorders (Table 2).

**Table 2:** **Comparison of prevalence of past illness or psychiatric disorders between use or non-use of faith healers(FHs)**

Variable	FH users (n = 383)	Control group (n = 424)	χ ^2^-value	p-value	Odds ratio (95% CI)
*History of past medical illness*					
Yes	155(40.5)	88(20.8)	37.2	<0.0001	2.6(1.9, 3.5)
No	228(59.5)	336(79.2)			1.0
*History of past psychiatric illness*					
Yes	89(23.2)	21(5.0)	57.1	<0.0001	5.8(3.5, 9.6)
No	294(76.8)	403(95.0)			1.0
*Presence of psychotic disorder*					
Yes	22(5.7)	0(0)*	25.0	<0.0001	27.0(3.6, 200.9)
No	361(94.3)	424(100)			1.0
*Presence of bipolar disorder*					
Yes	20(5.2)	3(0.7)	14.8	<0.0001	7.7(2.3, 26.2)
No	363(94.8)	421(99.3)			1.0
*Presence of depressive disorder*					
Yes	125(32.6)	61(14.4)	37.8	<0.0001	2.9(2.0, 4.1)
No	258(67.4)	363(85.6)			1.0
*Presence of anxiety disorder*					
Yes	71(18.5)	40(9.4)	14.1	<0.0001	2.2(1.4, 3.3)
No	312(81.5)	384(90.6)			1.0
Presence of other disorder**					
Yes	44(11.5)	3.8(3.8)	17.4	<0.0001	3.3(1.8, 6.0)
No	339(88.5)	408(96.2)			1.0

*By using continuity correction. **Alcohol- and substance-related disorders and eating disorders.

The stepwise multivariate binary logistic regression analysis of our data provides the independent factors of the participants and their associations with using FHs. The analysis shows that males were 4.8 times more likely than females to use FHs; those who were divorced or widowed were 3.5 and 4.8 times more likely, respectively, than singles to use FHs; those who were illiterate or held only primary levels of education were13.9 times more likely than those who held Masters or PhD degrees to use FHs; and those with lower incomes (<10,000SR) were 1.8 times more likely than others to use FHs.

Besides these socio-demographic characteristics, a history of past medical illness, past psychiatric illness, and presence of bipolar disorder or depressive disorder are independently associated with FH use (Table 3).

**Table 3:** **Independent factors associated with faith healer use (as determined by logistic regression analysis)**

Independent factor	Adjusted odds ratio (95% CI)
*Gender* (male)	4.8(2.9, 8.0)
*Marital status*	
Married	3.5(2.1, 5.9)
Divorced orwidowed	4.8(1.7, 13.6)
Single	1.0
*Education*	
Illiterate orprimary school	13.9(1.5, 128.8)
Intermediate orsecondary school	1.9(0.8, 4.7)
University ordiploma	0.54(0.22, 1.32)
Master or PhD	1.0
*Monthly Income* (<10000 SR)	1.8(1.1, 3.1)
*History past medical illness* (Yes)	1.7(1.1, 2.8)
*History of past psychiatric illness* (Yes)	4.8(2.1, 11.0)
*Presence of Bipolar disorder* (Yes)	6.1(1.1, 34.0)
*Presence of depressive disorder*(Yes)	2.6(1.5, 4.5)

## Discussion

As in the Saudi population [11], the participants were mostly young adults with at least an intermediate level of education. However, those with lower levels of education and lower incomes were more likely to use FHs. This fact indicates that people with more education and higher economic status may seek medical help instead of faith healing treatment. Al-Rowais et al. [12] completed a household survey in search of the reasons and health problems associated with seeking help from traditional healers. They reported the same inverse relationship between education and the likelihood of visiting a traditional healer; however, there was no association with income. Sorketti et al. [8] also reported that most of the visitors to the faith healing centers in Sudan were illiterate or held only basic primary education.

This may support the notion that users of FHs in developing countries generally have low educational and socioeconomic status. However, this is not the case in western communities where studies of sociodemographic correlates of the use of such type of healing in western communities resulted in inconsistent findings depending on the sample characteristics, type of healing intervention assessed, and time frame of the utilization question [2]. Considering epilepsy as a neurological disorder with major neuropsychiatric manifestations, we found that in some developing countries, such as Saudi Arabia and Pakistan and some minority ethnic communities in United Kingdom, epilepsy was frequently attributed to supernatural causes, and people with epilepsy sought treatment from FHs [13–15]. However, not only poor, less educated people, or those who cannot access the health care system,will seek treatment from FHs; well educated people and those with higher socioeconomic class may also seek help from FHs [16].

Females and singles were less likely to visit FHs. This contrasts with the findings of Al-Rowais et al. [12] and of Sayed et al. [5], who studied sociodemographic and clinical characteristics of FH users among psychiatric outpatients in Saudi Arabia. Both reported that the elderly and females were more likely to visit traditional healers. This could be explained by the restriction made by FHs and the reluctance of females to being interviewed by male interviewers in our study.

The FH users were more likely to report past medical or psychiatric histories. Compared to the control group, those with diagnosable psychiatric disorders were more likely to visit FHs. Before adjusting for confounding variables, this was especially true for those with either psychotic or bipolar disorders; however, after adjusting for confounding variables, it was especially true for those with bipolar or depressive disorders.

The prevalence of psychiatric disorders was higher among FH users; depressive and anxiety disorders being the most prevalent. Where few studies have studied those with psychiatric disorders in traditional healing settings, our finding of a high prevalence of psychiatric disorders is consistent with what has been reported in the literature among those with various cultural and ethnic backgrounds. In spite of the consistency of association with psychiatric disorders, the most prevalent disorder varies between studies. The differences can be explained by use of different study procedures including diagnostic instruments and study settings. Two studies reported by Abbo et al. [17, 18] and Sorketti et al. [8] were conducted in facilities that provided overnight accommodations for the visitors. This may explain the high prevalence of severe psychiatric disorders, (i.e., psychotic and bipolar disorders) in their reports. Abbo et al. reported that 60.2% of those who used traditional healers in Uganda had a diagnosable psychiatric disorder. They also found that psychotic depression, mania, and schizophrenia were the most frequently observed disorders among their participants. Sorketti et al. reported that the most prevalent diagnosis for those under treatment in traditional healer centers in Sudan waspsychotic disorders (34.6%), manic episodes (27.4%), andmajor depressive disorders (15.8%).

As in our study, studies reported by Saeed et al. [19] and Ngoma et al. [20] recruited participants from facilities that did not allow patients to stay overnight; rather, they provided outpatient care. Both reported a high prevalence of depressive and anxiety disorders. Similar to our findings, Saeed et al. reported that, in rural Pakistan, 61% of FH users had psychiatric disorders, including a high proportion who had major depressive episodes (24%) or generalized anxiety disorders (15%). Ngoma et al. reported that the prevalence of psychiatric disorders among users of traditional healers in Tanzania was double (48%) that of primary care patients (24%). They also found that mixed anxiety-depressive disorder was the most prevalent (27.8%) among those who used traditional healers.

While our study has many advantages, we acknowledge a number of limitations. Because of the use of convenience sampling, our results should be cautiously generalized and should not be regarded as representative to all patients in Saudi Arabia. Also, the control group was recruited from visitors to shopping malls; therefore, they may not represent the general population. We chose this method because at this time, no epidemiological study has investigated the prevalence of psychiatric disorders in the general population of Saudi Arabia. Another limitation is our use of a self-report scale; therefore, recall bias cannot be excluded. Finally, the use of general questions about income may not precisely reflect the social class of the participants.

## Conclusion

We showed that having a psychiatric history or a current psychiatric disorder are factors associated with FH use, and that a high percentage of FH users had a diagnosable psychiatric disorder. This study may complement other studies performed in Saudi Arabia that focused on studying the practices of FHs [6, 21] and the psychosocial characteristics of FH users [5, 12, 22]. We also investigated the psychiatric patients within the faith healing system. This study is likely to improve our understanding of the psychosocial correlates of visiting FHs and the types and prevalence of psychiatric disorders among those who use faith healing. Further research should assess how to facilitate access to the mental health care system for those who use FHs.
